# Supplementary figures and images for: Maturity2, a novel regulator of flowering time in Sorghum bicolor, increases expression of SbPRR37 and SbCO in long days delaying flowering
Source: PLoS One. 2019 Apr 10;14(4):e0212154. doi: 10.1371/journal.pone.0212154 (PMC6457528; doi:10.1371/journal.pone.0212154)

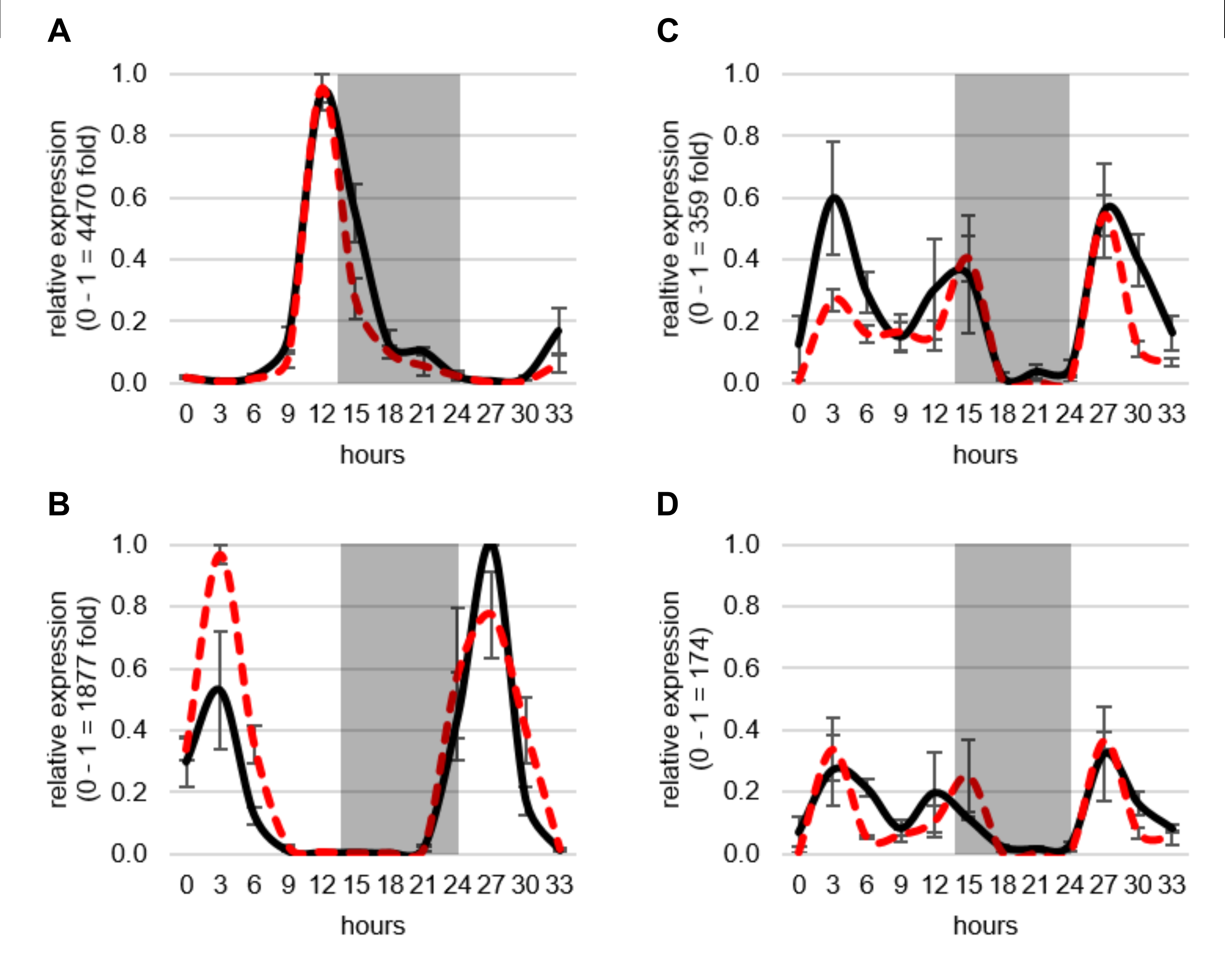

Supplement: S1 Fig — There were no consistent differences in expression of (A) SbTOC1, (B) SbLHY, (C) SbGhd7, and (D) SbEhd1 between 100M (solid black line) and 80M (dashed red line). (TIF) [file pone.0212154.s001.tif]

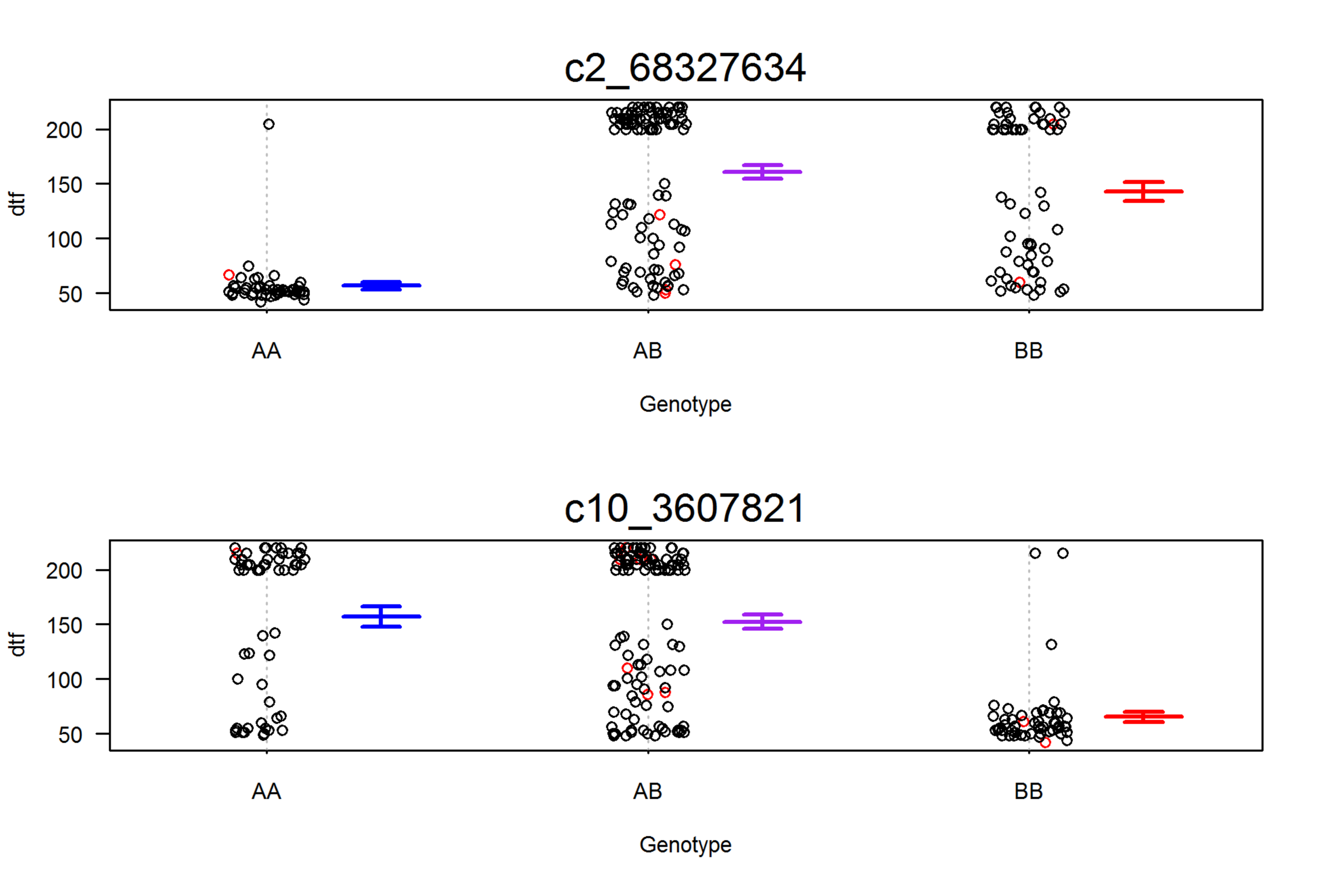

Supplement: S2 Fig — Recessive alleles of Maturity genes contribute to earlier flowering. 80M (AA) is recessive for ma2, while Hegari (BB) is dominant. Individuals genotyped AA for the QTL on SBI02 (represented by marker c2_68327634) flowered ~100 d earlier than those genotyped BB. 80M is dominant for Ma4, and individuals genotyped AA at the QTL on SBI10 (represented by marker c10_3607821) flowered ~100 d earlier than those genotyped BB. (TIF) [file pone.0212154.s002.tif]

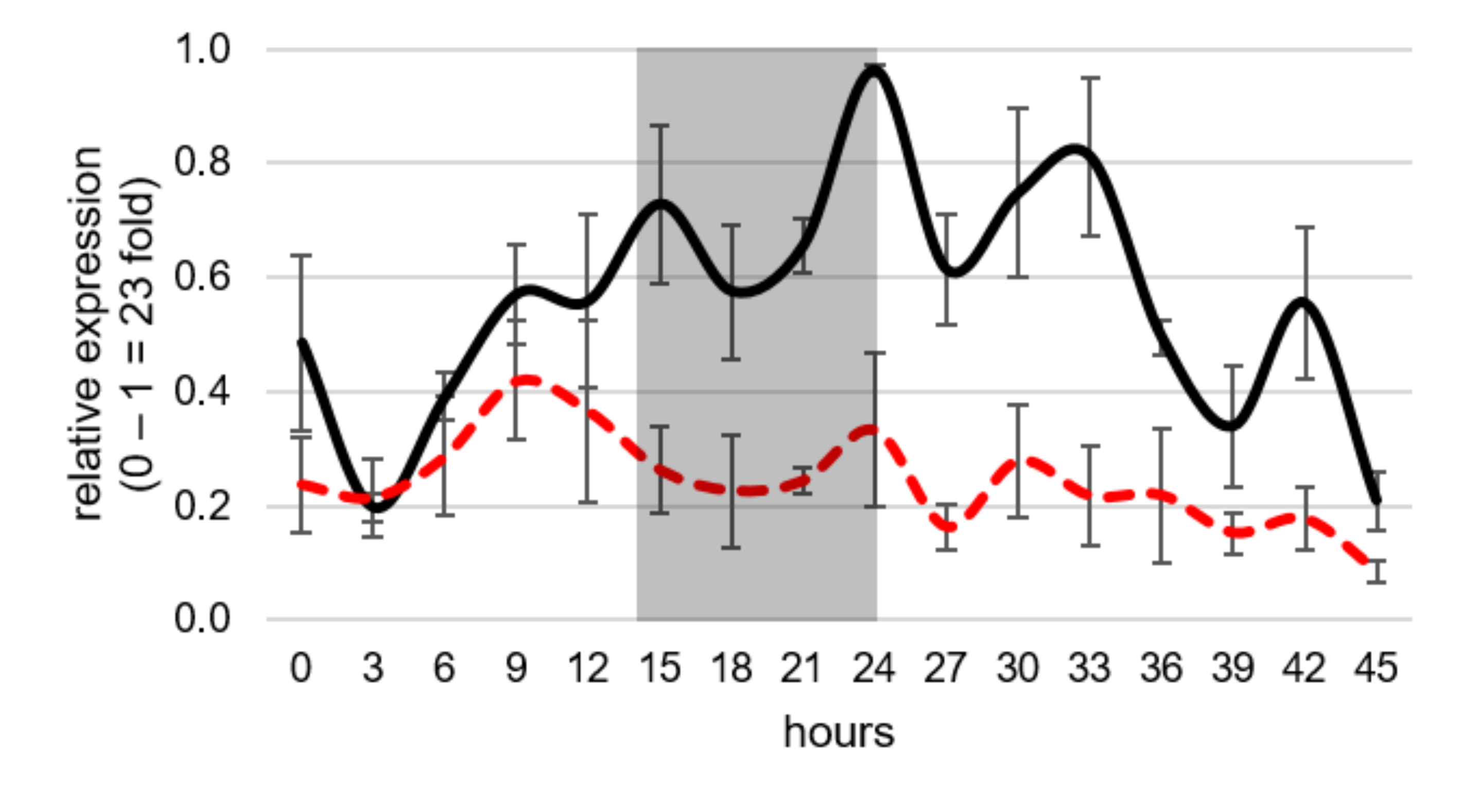

Supplement: S3 Fig — The expression of Sobic.002G302700 does not cycle diurnally in 100M (solid black line) or 80M (dashed red line). There was no difference in expression between 100M and 80M in the first day. Expression was slightly elevated in 100M compared to 80M during the night and through the following morning. (TIF) [file pone.0212154.s003.tif]
