# Supplementary material for: Maturity2, a novel regulator of flowering time in Sorghum bicolor, increases expression of SbPRR37 and SbCO in long days delaying flowering
Source: PLoS One. 2019 Apr 10;14(4):e0212154. doi: 10.1371/journal.pone.0212154 (PMC6457528; doi:10.1371/journal.pone.0212154)
Supplement: S1 Table — (DOCX) [file pone.0212154.s004.docx]

**Table S1. Ma_2_ (Sobic.002G302700) sequencing and qPCR primers**

| Type |  | Primer sequence |
| --- | --- | --- |
| Template amplification | F | GCGTCATGCTATGTTTCAGCC |
|  | R | CATCCTGCTGCATAAGCTCC |
| Sanger sequencing | F | ATGCATCAATTGTTTGGTACAGA |
|  | R | TTGATTCACGTCTGCATGCTT |
| qPCR | F | TCGGGCACTTGGAATTTGAGA |
|  | R | TGCTGTGCGCAACAAAATTAGA |
